# Development of a Validated HPLC/Photodiode Array Method for the Determination of Isomenthone in the Aerial Parts of *Ziziphora tenuior* L.

**DOI:** 10.17795/jjnpp-12504

**Published:** 2013-11-02

**Authors:** Nasrollah Ghassemi, Mustafa Ghanadian, Lili Ghaemmaghami, Haran Kiani

**Affiliations:** 1 Department of Pharmacognosy, School of Pharmacy, Isfahan University of Medical Sciences, Isfahan, IR Iran; 2 Department of Biology, Faculty of Science, University of Isfahan, Isfahan, IR Iran; 3 Isfahan Pharmaceutical Sciences Research center, Isfahan University of Medical Sciences, Isfahan, IR Iran

**Keywords:** Isomenthone, Chromatography, High Pressure Liquid, Gas Chromatography-Mass Spectrometry

## Abstract

**Background:**

*Ziziphora tenuior* L. known as Kakuti in Persian, is used in traditional medicine for fever, dysentery, uterus infection and as an analgesic. It is used also in the treatment of gastrointestinal disorders as carminative, or remedy of diarrhea or nausea. Major components of plant essential oil including pulegone, isomenthone, thymol, menthone, and piperitone are suggested to be responsible for the mentioned medicinal properties.

**Objectives:**

In the present study, a normal high performance liquid chromatography (HPLC)/photodiode array validated method for quantification of isomenthone, one of the major constituents of *Ziziphora,* was established for the first time with a simple, rapid and accurate method.

**Materials and Methods:**

HPLC analysis was done on a Waters system, equipped with 515 HPLC pump and waters 2996 photodiode array detector. The column was a Nova-Pak Silica (3.9 × 150 mm), and Empower software was used for the determination of the compounds and processing the data. The method was validated according to USP 32 requirements.

**Results:**

A selective method for the resolution of isomenthone from two nearest peaks, thymol, and carvacrol was obtained with gradient system of hexane (A), and hexane: ethyl acetate (9:1) (B), starting with A: B (100:0) for 2 minutes, then 0−20% B in 5 minutes, A:B (80:20) for 5 minutes, then 20-30% B in 3 minutes, 30-100% B for 5 minutes, A:B (0:100) for 4 minutes following with equilibrating for 10 minutes. The flow rate was 1 mL/min at 22˚C and the injection volume for the standards and the samples was 20 μL. The retention time for isomenthone was found to be 7.45 minutes. The regression equation was y = 143235x - 2433 with the correlation co-factor R^2^ = 0.9992 and the percent recovery of 65.4 ± 3.85%. The sample obtained from 5 g of *Z. teniour* dried powder in 6 mL extract was standardized to contain 1.14 ± 0.030 μL/mL isomenthone which is equivalent to % 1.37 μL/g of the dried powdered plant. Limit of detection (LOD) and Limit of Quantification (LOQ) were 0.037, and 0.122 µL/mL determined by using the formula based on the signal to noise ratio.

**Conclusions:**

Due to this fact that plant extracts may cause irreversible damages to the capillary GC columns, using validated HPLC method for the analysis of these compounds in cruse plant extracts is recommended.

## 1. Background

For centuries, herbal medicine was the only available source in drug treatment system. Despite remarkable progress and development of synthetic drugs, medicinal herbs and derived natural products still have a thriving market (1). They are also used in food formulations as additives to promote health (2). But, some of the herbal product shops do not use acceptable standards or even, medicinal herbs might be supplied without standardization of the constituents responsible for the claimed therapeutic effects. Therefore, non-standard types of plants are supplied instead of the standard ones. In many countries, there is a great risk to human health because self-medication or self-treatment by herbal remedies which is very common. So, the control of herbal ingredients in starting materials and final products is a necessity to attenuate adulterations or avoid unwanted adverse effects. One of the frequently reported adulterations of the plants of the mint family is related to *Ziziphora* genus. *Ziziphora* genus (Lamiaceae) is consisted of four species of annual and perennial herbaceous species, namely *Z. clinopodioides*, *Z. tenuior* L., *Z. persica* Bunge and *Z. capitata* L (3). The adulteration of this genus is frequently reported with *Zataria multiflora* (Shiraz thyme), *Thymus kotschyanus*, and *Thymus vulgaris* (garden thyme). *Ziziphora* or Kakuti in Persian traditional medicine is ascribed to dried aerial parts of *Ziziphora tenuior* L which contains at least 1.2% of the essential oil (volume/weight) (4). This plant is distributed widely in Iran, Turkmenistan, Afghanistan, Armenia, Anatolia, Pakistan, Central Asia, Syria, and Transcaucasia (3). It is a herbaceous, annual plant, with 5-15 cm slender stems, linear lanceolate leaves, elongated and compact spike inflorescence, short pedicel 1.5-4 mm, and sparsely hairy purple or green calyx (4). *Ziziphora* is used in traditional medicine to treat fever, dysentery, uterus infection and analgesic (5). It is used also in the treatment of the gastrointestinal disorders as carminative, or remedy of diarrhea or nausea (6, 7). Major components of its essential oil are pulegone, isomenthone, thymol, menthone, and piperitone (7-10). These compounds are suggested to be responsible for the mentioned medicinal properties (11-13). So, these contents can be quantified and applied as an important index in the quality evaluation or even the detection of the bulk drug. According to pharmacopeias guide line, before marketing the plant drugs, their spectroscopic profile and phytochemical pattern are required for authentication and control (14). To the best of our knowledge, no studies on the quantitative HPLC determination of isomenthone were reported in the literature.

## 2. Objectives

In this paper a high performance liquid chromatography (HPLC) validated method for isomenthone as one of the major constituents of *Ziziphora* was established for the first time with a simple, rapid and accurate method.

## 3. Materials and Methods

### 3.1. Plant Material

The aerial parts of *Ziziphora tenuior* L. (Labiatae) were collected during its flowering stage in May 2012 from the Baharestan in Isfahan, Iran and was identified by Dr. L. Ghaemmaghami, Department of biology, Faculty of Science, University of Isfahan. A herbarium specimen No. 1514 is deposited in the herbarium of the Faculty of Pharmacy, Isfahan University of Medical Sciences (Iran) for future reference.

### 3.2. Instrumentation

HPLC (High-performance liquid chromatographic) analysis was done on a Waters system, equipped with 515 HPLC pump, waters 2996 photodiode array (PDA) detector (Waters, Milford, MA, USA). The column was a Nova-Pak Silica, 3.9 × 150 mm (Waters, Milford, MA, USA) and Empower software was used for the determination of compounds and in processing the data.

### 3.3. Chemicals

Methanol, dichloromethane, ethyl-acetate, and hexane for extraction were of analytical grade purchased from Merck Company (Germany). Hexane and ethyl acetate HPLC-grade solvents were purchased from Caledon Company (Canada). Isomenthone, as a standard, was purchased from Roth (Karlsruhe, Germany).

#### 3.3.1. TLC Analysis

The extracts, or essential oil was chromatographed on TLC, silica gel 60 F254 plates (Merck, Germany). Using the Co-TLC method with the standards thymol, pulegone, carvacrol, and isomenthone, the sample was applied on TLC (silica gel G60 F254) with the solvent system of Hexane: ethyl acetate (97:3). After developing and drying, the plates were sprayed using a vanillin reagent (1 g vanillin, 100 mL ethanol, 5 mL sulfuric acid) which is adequate for identification of essential oils.

#### 3.3.2. GC/MS Analysis 

Determination of the *Z. teniour* essential oil was done on the Agilent 7890 A coupled Mass detector with injection volume, 0.1 μL, HP-5 MS capillary column (30 m× 0.25 mm; film thickness: 0.25 μm); carrier gas, He; flow rate, 2 mL/min; injector temperature, 250˚C; temperature program, 60-275˚C at 4˚C/min. The mass spectra was obtained with electronic impact, ionization potential 70 eV, ion source temperature 250°C, ionization current 1000 μA, resolution 1000 and mass range 30-300. Identification of the compounds based on their mass spectra was done using the library database Wiley 275 software. The retention indices (RI) were calculated with reference to the n-alkane series injected in the current temperature programmed run, before analysis of the real sample (14, 15).

### 3.4. Determination of Isomenthone

Using high pressure liquid chromatography, isomenthone is determined through external standard calibration method.

### 3.5. Solvent Effect

In order to evaluate the solvent effects as one of the affecting parameters on the extraction efficiency and distribution coefficient of the isomenthone, hexane, dichloromethane, ethyl acetate, and methanol with different strength and selectivity’s were selected.

### 3.6. Extraction Method Optimization

Two extraction methods which consisted of hot solvent extraction by rotary instrument operating at 20 rpm, under the air pressure and the temperature of 50˚C (60 minutes), and ultrasonic radiation (60 minutes) were compared to check the effect of the procedure on the extraction.

### 3.7. Sample Preparation

In a volumetric flask, 5 g of the plant powder was weighed and 25 mL of the solvent were added to the sample powder. It was extracted with a rotary for 60 minutes. The obtained mixture was filtered, and washed three times with 10 mL of the solvent. Using the rotary evaporator under vacuum and at a 40˚C temperature, the filtrates were combined and evaporated till dryness. Rotary evaporated extract was washed 3 times with 6 mL HPLC-grade hexane.

### 3.8. Preparation of the Standard Solutions

Form pure liquid isomenthone, 10 μL was diluted in 2 mL of HPLC-grade hexane to prepare the stock solution of 5μL/mL. Different standard solutions were prepared by serially diluting the stock solution to concentrations of 0.25, 0.5, 0.75, and 1 μL/mL.

### 3.9. Validation

The reliability of the HPLC-method for analysis of isomenthone was validated through its linearity, reproducibility, repeatability, and recovery (16-18).

### 3.10. Selectivity

**Figure 1.**
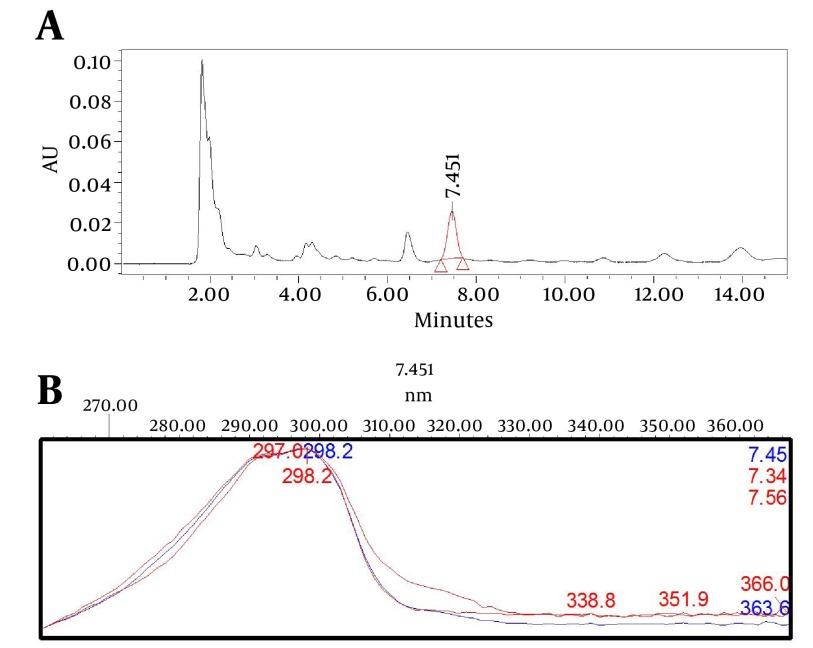
A. The Chromatographic Signal of Isomenthone in the Z. teniour Ethyl Acetate Extract. B. Peak Purity Matches of Isomenthone in Three Different Points at the Middle, Left, and Right Sides of the Isomenthone Peak (7.34, 7.45, and 7.56 Minutes) Did not Show Significant Impurities in the Isomenthone Peak (Impurities < 0.05).

After developing the separation method, using a photodiode array detector and a peak purity processing option in the Empower HPLC software, the target component peak (isomenthone) in the real sample was evaluated for peak purity and resolution. The purity was checked through peak purity matches of three point in the middle, right and left sides of the isomenthone peak (Figure 1). It was done to check the presence of possible impurities and the ability of the method to accurately measure the component of interest (19).

### 3.11. Limit of Detection and Limit of Quantification

Limit of detection (LOD) and limit of quantification (LOQ) were calculated using equations, LOD = 3 × S/N and LOQ = 10 × S/N. USP 35-NF 30 defines S/N = 2h/hn, where h is the height of the peak corresponding to the component interest from the peak apex to the midpoint of the noise, and hn is the difference between the largest and smallest background noise value observed over a distance equal to five times the width at the half-height of the peak of interest. The USP definition from S/N is useful in the gradient systems, in which a drift in the baseline is present. Otherwise, in the case of using signal to noise ratio, noise will be overestimated when drift is present (17).

### 3.12. Linearity and Range

Recommended range to be examined in validation studies start with limit of quantification (LOQ) and extend up to beyond the expected target measurement level. The linearity means how well a calibration plot of response versus concentration approximates a straight line. It was done by linear squares regression. Correlation coefficient (r^2^) in addition to plot slop and intercept provided desired information on linearity. A linearity correlation coefficient greater than 0.999 is considered to be acceptable (18).

### 3.13. Precision

Precision is the degree of agreement among individual test results, when a series of determinations for the same analyte are done repeatedly. It could be evaluated through repeatability assays like intra-day precision, and intermediate precision assays like inter-day precision followed by determination of relative standard deviation (RSD). RSD is calculated using the equation RSD, % = (SD/X) * 100, where SD is the standard deviation, and X is the mean of responses. For HPLC of a major component in the in-vitro systems, RSD less than 3% is required (18).

#### 3.13.1. Intra-day Precision

It depends on instrumental precision and is done through sequential, repetitive injection of the same sample within the selected range. Intra–day precision was determined by injecting three replications of 4 different concentrations (0.125, 0.5, 0.75, 1.00, and 1.25 μL/mL), three times in the same day (n = 9). Peak area was measured and RSD% was calculated (18).

#### 3.13.2. Inter-day Precision

Inter-day precision was determined by injecting of three replicates of 4 different concentrations (0.125, 0.5, 0.75, 1.00, and 1.25 μL/mL) on three consecutive days in a week (n = 9). Peak area was measured and RSD% was calculated (18).

### 3.14. Recovery

Recovery studies are required to check the accuracy of an analytical method. It guarantees that the real quantification of target components is measured. The determination of this parameter was performed by spiking the standard in different levels of isomenthone into a blank matrix lacking isomenthone. The concentrations are better to cover the selected range, one in the middle of the range and another near the end of the calibration curve. Confirming through GC-MS, *Zataria multiflora Boiss* lacking isomenthone was selected as the blank sample matrix for recovery tests. Then, three portions and each five grams of plant powder was weighed accurately, and added to a volumetric flask. To the sample powder, 25 mL of ethyl acetate was added as the solvent. One part was selected as blank for the background, and the two others spiked with 1 mL of isomenthone standard of 5 μL/mL, and 10 μL/mL. The samples were extracted as mentioned in the sample preparation method, and volumized to 10 ml. In each additional level, three determinations were carried out and the recovery percentage was calculated in every case (18, 19).

### 3.15. Statistical Analysis

Data acquisition and analysis were performed using Waters Empower chromatography software (Build 1154, Waters Corporation, Milford, USA). Data were reported as Mean ± SD and the results were analyzed statistically by Excel 2003 software.

## 4. Results

Using GC-MS analysis and Co-TLC with standards, the identified volatile constituents in addition to their relative percentages based on total ion count in the essential oil of the aerial parts of *Z. teniour* are listed in Table 1. As it is clear, isomenthone, pulegone, thymol, and piperitone were the main volatile constituents.

**Table 1.** Volatile Components of the Aerial Part Essential oil of *Ziziphora teniour * L.

Components	RI ^a^	TIC, %	Identification Method
**Isomenthone **	1164	7.15	RI-MS-TLC
**L-menthone**	1165	< 1.00	RI-MS
**Pulegone**	1238	33.47	RI-MS-TLC
**Piperitenone**	1252	3.95	RI-MS
**Carvacrol**	1255	< 1.00	RI-MS
**Thymol**	1290	14.51	RI-MS-TLC
**Piperitone**	1338	40.93	RI-MS

^a^ Abrreviations: RI, retention indices.

The HPLC method carried out in this study was aimed to develop a chromatographic system, capable of eluting, resolving, and quantification isomenthone in crude plant materials. The preliminary investigations were directed toward evaluating the effect of various factors on the system. The PDA spectra of isomenthone showed that the compound absorbs appreciably at 298 nm which was selected as the detection wave length in liquid chromatography (Figure 1). In comparison between the normal and reverse columns, the best separation efficiency was obtained by using the normal silica column. Isomenthone is a nonpolar terpenoid, with large retention factor in c-18 columns, which leaded to wide peaks. The aim of a good separation is sharp and symmetrical peaks which depend to peak band width. Resistance to mass transfer due to the possible Van der waals forces between C-18 side chain and isomenthone which is a nonpolar compound and lack of hydrogen bond forces between this compound and aqueous mobile phase in reverse system leads to increased band width according to van deemter formula. So silica column with more resolution and sharp peaks is selected for isomenthone. Other affecting factors on resolution are mobile phase composition, temperature, isocratic or gradient criteria. Mobile phase investigations showed that hexane/ethyl acetate with UV cut off far from standard UV maximum absorption at 298 nm are suitable, but the ratio of hexane or ethyl acetate in the mobile phase was the key to a good separation. The best selectivity factor for resolution of isomenthone from two nearest peaks, thymol, and carvacrol was obtained with a gradient mobile phase system of hexane (A), and hexane: ethyl acetate, 9:1 (B). It was started with an injection program with A: B (100:0) for 2 minutes, then 0−20% B in 5 minutes, A:B (80:20) for 5 minutes, then 20-30% B in 3 minutes, 30-100% B for 5 minutes, A:B (0:100) for 4 minutes followed with equilibration for 10 minutes. The flow rate was 1 mL/min at 22˚C and the injection volume for standards and samples was 20 μL. The retention time for isomenthone was observed to be 7.45 minutes.

After optimizing the mobile phase, and the column, between the application of a UV/Vis Detector dual-wavelength or UV/Vis photodiode array detector, the later was selected due to the option of spectral matching and peak purity checking in Diode Array Detection in high performance liquid chromatography. In order to check the selectivity of the method, after developing the separation method, using photodiode array detector and peak purity processing option in the Empower HPLC software, isomenthone peak of the sample and standard chromatographs were similar and applying the option of peak purity matches in the program on isomenthone peak did not show impurities (Figure 1). In selecting the best solvent in the extraction method, extraction with methanol, ethyl acetate, dichloromethane, and hexane as most frequent and general solvent with good selectivity for isomenthone were compared. As cleared from Table 2, based on the target compound recovery methanol and ethyl acetate showed better recovery, but among them ethyl acetate was selected due to its better selectivity and recovery (Table 2). The solubility behavior of isomenthone which is a nonpolar monoterpene gives us the key to the choice of the solvent. Theoretically, methanol with more Hildebrand parameter should have better solubility characteristics, but because of higher hydrogen bonds in methanol than ethyl acetate, thus it is not as good as ethyl acetate in salvation of nonpolar monoterpenes. The reason for this is the type of dispersion forces, and lack of polar, and hydrogen bonding forces between the molecules of oily monoterpenes like isomenthone. After selecting ethyl acetate as the extraction solvent, for optimizing the extraction method, hot solvent extraction with rotary was compared with ultra-sonication method. Hot solvent extraction of the powder plant (5 g, 60 min, 50˚C) with ethyl acetate was selected as the best method for isomenthone analysis (Table 2).

**Table 2.** The Effect of Solvents and Extraction Procedures on the Determination of Isomenthone in *Z. tenuior *

Sample	Extraction method		Solvent			
	Hot solvent	Ultrasonication	Methanol	Ethyl acetate	Dichloromethane	Hexane
***Z. tenuior***	1.14 ± 0.041	0.79 ± 0.015	1.09 ± 0.039	1.14 ± 0.041	0.85 ± 0.034	0.76 ± 0.028

**Figure 2.**
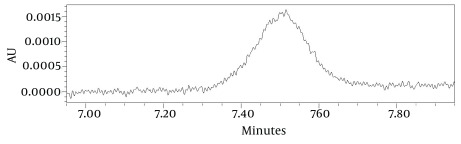
Limit of Detection (LOD), and Limit of Quantitation (LOQ) of Isomenthone (0.125 µL/mL) HPLC Chromatogram at 298 nm. Using USP 32 Defintion of S/N = 2h/hn, the LOD and LOQ Were Calculated as 0.037 µL/mL and 0.122 µL/mL

Using USP 35-NF 30 definition of signal to noise ratio: S/N = 2h/hn, the LOD and LOQ were calculated as 0.037 µL/mL and 0.122 µL/mL for isomenthone, respectively (Figure 2). LOQ of 0.122 μL/mL was the minimum concentration of isomenthone in the sample which was quantified experimentally with acceptable precision (RSD < 3). Low molar UV absorption of monoterpenes prevents us from the detection or the quantification of lower amounts of isomenthone. Using isocratic mobile phase system or gas chromatography with FID detector may reduce the LOD and LOQ values. The linear relationship between the detector response in electron volt for area of the target peak, and different concentrations of isomenthone was certified in the range of LOQ to beyond target level in the real sample according to 0.125 to 1.25 μL/mL, with a correlation coefficient (R^2^ square) of 0.9992 and equation formula of y = 143235x - 2433 (Table 3).

**Table 3.** Linearity, LOD, and LOQ parameters of Isomenthone analysis in *Z. tenuior *

Compound	LOD	LOQ	R^2^ Square	Equation	Linear Range, μL/mL
**Isomenthone**	0.037	0.122	0.9992	y = 143235x-2433	0.125-1.25

The instrumental precision by intra-day results and intermediate precision through inter-day results of repetitive isomenthone quantitation in different concentrations in the selected linear range, 0.125 to 1.25 μL/mL, and in the real sample as is presented in Table 4, were obtained with RSD < 3 which was acceptable.

**Table 4.** Repeatability and Intermediate Precision Assays of Standards and Real Sample (n = 9)

Sample, μL/mL	intra-day, Mean ± SD	intra-day, RSD, %	inter-day, RSD, %
**Isomenthone 0.125**	15314 ± 404	2.64	2.80
**Isomenthone 0.25**	31520 ± 753	2.39	2.67
**Isomenthone 0.50**	70406 ± 1732	2.46	2.06
**Isomenthone 0.75**	106223 ± 1275	1.20	1.41
**Isomenthone 1.00**	142812 ± 3156	2.21	2.34
**Isomenthone 1.25**	174165 ± 3622	2.08	2.32
**Real sample (*Z. teniour*)**	160854 ± 4230	2.63	2.81

The accuracy of the validated method describes the extent to which test results quantified by the method and the real amount. In this method, accuracy was evaluated as recovery test after spiking of 1 mL of isomenthone standard at two levels of 5, and 10 μL/mL standard to the *Zataria multiflora *powder plant as blank sample matrix, and was found to be 65.4 ± 3.85 (Table 5).

**Table 5.** Recovery Test of Isomenthone by Spiking Different Levels of Standard into Blank Matrix a

Spiked Standard, μL/mL	Found, μL/mL	Mean Recovery, % (n = 3)	Total Recovery, %
**0.0**	-	-	65.4 ± 3.85
**0.5**	0.34 ± 0.038	68.1 ± 4.2	
**1.0**	0.62 ± 0.061	62.7 ± 3.5	

^a^Mean ± SD

## 5. Discussion

**Figure 3.**
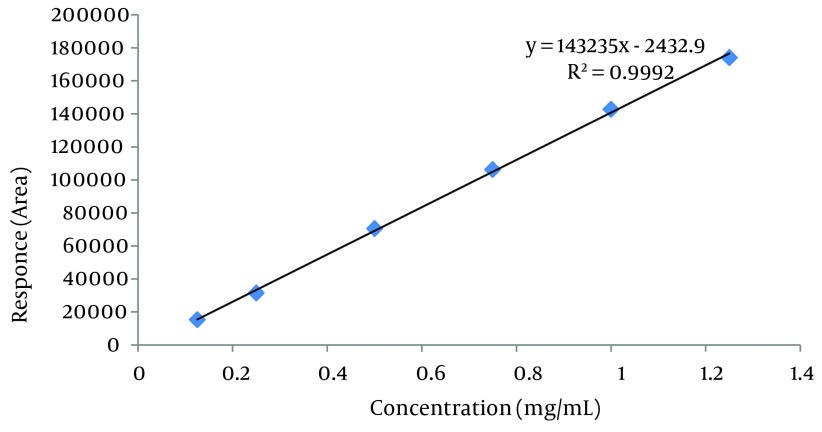
Calibration Curve of Isomenthone at 298 nm Using the Empower processing software, the calibration curve was determined by linear regression in the range of 0.125-1.25 µL/mL. The regression equation was y = 143235x - 2432.9, where X is the concentration of isomenthone in sample (µL/mL) with the correlation co-factor (R^2^) of 0.9992.

The retention time for isomenthone was found to be 7.45 minutes. By the aid of the Empower and Excel software, the calibration curve was determined by linear regression in the range of 0.125 to 1.25 µL/mL. The regression equation was y = 143235x - 2433, where X is the concentration of isomenthone in the sample (µL/mL) with the correlation co-factor R^2^ = 0.9992 and the percent recovery was 65.4 ± 3.85%. The sample obtained from 5 g of *Z. teniour* dried powder in 6 mL extract was standardized to contain 1.14 ± 0.030 μL/mL isomenthone which is equivalent to % 1.37 μL/g of dried powdered plant. Limit of detection (LOD) and limit of quantification (LOQ) were 0.037, and 0.122 µL/mL determined by using the formula based on the signal to noise ratio (Figure 3).

The results from the validation of the newly established method for the determination of isomenthone as linearity, selectivity, precision, repeatability, reproducibility and accuracy showed that the proposed method is selective, and reliable. This method could be a good alternative to the already GC methods for identification of these compounds in the plant extracts. In gas chromatography (GC), even after filtering or careful extraction procedures, small nonvolatile materials are present in the injected sample. So, due to this fact that plant extracts may cause irreversible damages to the capillary GC columns, using validated HPLC method for the analysis of these compounds in cruse plant extracts is recommended.
